# Myocardial protection in cardiac surgery: a comprehensive review of current therapies and future cardioprotective strategies

**DOI:** 10.3389/fmed.2024.1424188

**Published:** 2024-06-19

**Authors:** Pascal Chiari, Jean-Luc Fellahi

**Affiliations:** ^1^Service d’Anesthésie Réanimation, Hôpital Universitaire Louis Pradel, Hospices Civils de Lyon, Lyon, France; ^2^Laboratoire CarMeN, Inserm UMR 1060, Université Claude Bernard Lyon 1, Lyon, France

**Keywords:** cardiac surgery, ischemia–reperfusion injury, myocardial protection, preconditioning, postconditioning

## Abstract

Cardiac surgery with cardiopulmonary bypass results in global myocardial ischemia–reperfusion injury, leading to significant postoperative morbidity and mortality. Although cardioplegia is the cornerstone of intraoperative cardioprotection, a number of additional strategies have been identified. The concept of preconditioning and postconditioning, despite its limited direct clinical application, provided an essential contribution to the understanding of myocardial injury and organ protection. Therefore, physicians can use different tools to limit perioperative myocardial injury. These include the choice of anesthetic agents, remote ischemic preconditioning, tight glycemic control, optimization of respiratory parameters during the aortic unclamping phase to limit reperfusion injury, appropriate choice of monitoring to optimize hemodynamic parameters and limit perioperative use of catecholamines, and early reintroduction of cardioprotective agents in the postoperative period. Appropriate management before, during, and after cardiopulmonary bypass will help to decrease myocardial damage. This review aimed to highlight the current advancements in cardioprotection and their potential applications during cardiac surgery.

## 1 Introduction

Cardiac surgery with cardiopulmonary bypass (CPB) includes an expected ischemia–reperfusion (I/R) sequence, leading to myocardial injury and significant postoperative morbidity and mortality. The unavoidable and sustained elevation of postoperative serum troponin is multifactorial because of direct surgical trauma, systemic inflammation, and reversible ischemia or irreversible necrosis. Its short- and long-term prognosis value has been validated for years (1–3). Irrespective of the surgical procedure itself, the early or late elevation of peak serum troponin I has probably a distinct meaning, with a pejorative value in cases of elevation beyond the 24th postoperative hour (4, 5). A large prospective cohort study recently demonstrated that threshold values of hypersensitive troponin I above 5,670 ng/L on postoperative day 1 after coronary artery bypass grafting (CABG) or aortic valve surgery and above 12,981 ng/L following other cardiac surgeries were associated with increased mortality at day 30 (6). A whole literature on myocardial protection techniques during cardiac surgery has therefore emerged. While cardioplegia remains the cornerstone of intraoperative cardioprotection, the lack of consensus on its practice is striking. The concept of cardioplegia is based on the administration of a hyperkalemic solution that induces a rapid diastolic arrest by depolarizing cardiomyocytes. Different solutions and components (crystalloids, warm or cold blood cardioplegia, anterograde, retrograde, or combined delivery approaches) are used by surgical teams and make synthesis difficult (7). It is beyond the scope of this review to provide a detailed overview of the different cardioplegia solutions and practices. In this update, we will focus on other cardioprotection approaches administered during cardiac surgery that have been developed in addition to cardioplegia techniques.

## 2 The concept of preconditioning and postconditioning

**Figure 1:**
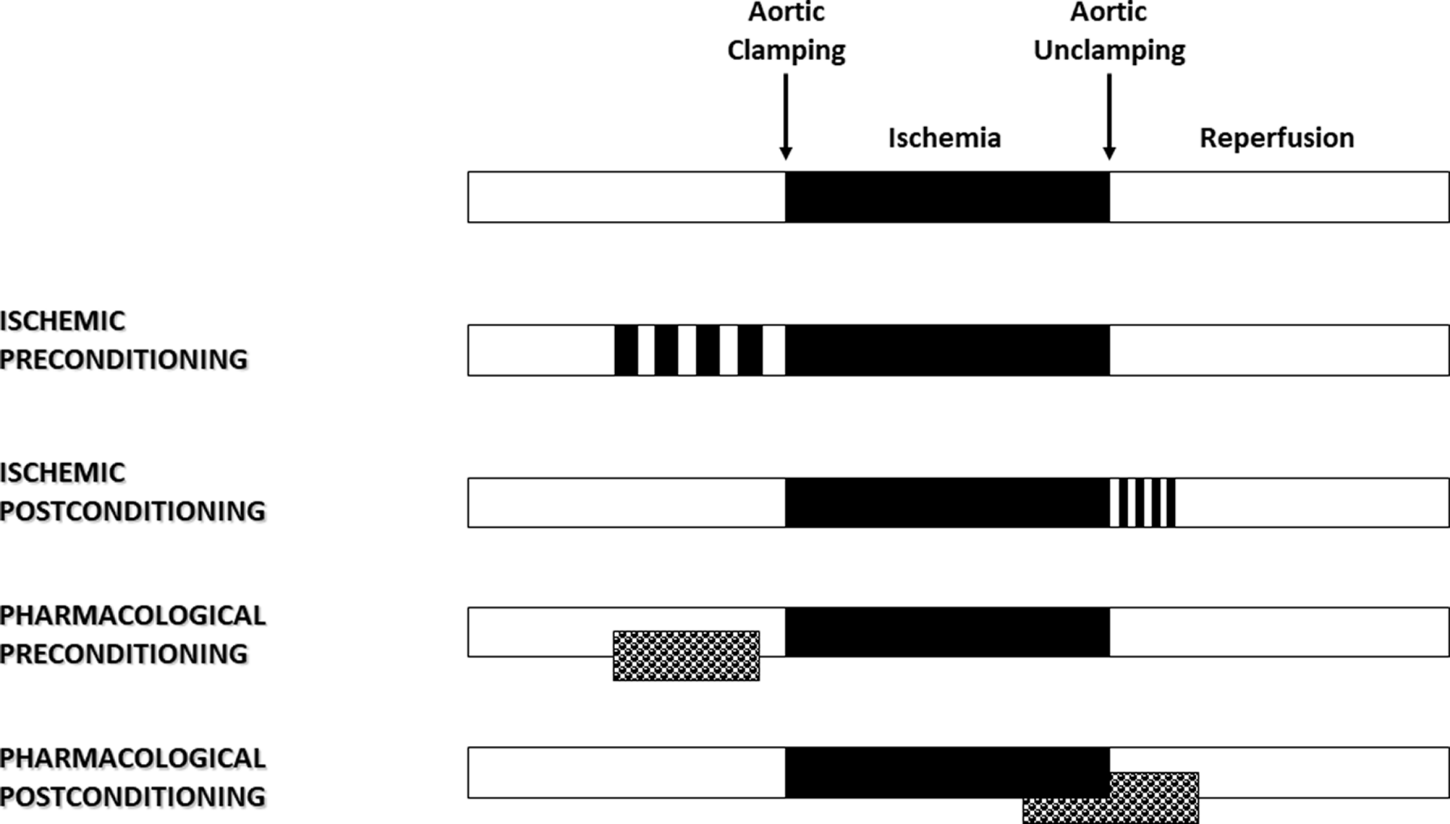
Ischemic and pharmacological preconditioning and postconditioning.

In 1986, Murry et al. (8) published an experimental study that had a major impact and proved to be a turning point in the field of cardioprotection. Using an *in vivo* animal model, they demonstrated that four brief sequences of 5 min I/R, applied just before a lethal 40-min ischemia, reduced the size of myocardial infarction by more than 75% (Figure 1). The ischemic preconditioning concept was born. Then, we learned that patients presenting with an inaugural myocardial infarction have greater myocardial damage than those whose infarction is preceded by a period of angina (9). Shortly after, Ishihara et al. (10) demonstrated that prodromal angina occurring before the onset of infarction had a beneficial effect on long-term prognosis. The human myocardium is therefore just as receptive as the animal myocardium to the preconditioning signal. It is possible to modify the phenotype of the myocardium before an ischemic process so that it increases its tolerance to oxygen deprivation. By shifting the time-dependent necrosis curve to the right, preconditioning reduces the lesion for a given time.

Myocardial reperfusion, while essential to limit the infarct size, can itself induce injury, thereby reducing its expected beneficial effects (11). It is described as a double-edged sword. Several abrupt metabolic and biochemical changes occur within minutes of reperfusion, including the generation of reactive oxygen species (ROS), intracellular calcium overload, and rapid restoration of normal intracellular pH. In 2003, Zhao et al. (12) demonstrated that the application of brief I/R sequences in the early stages of reperfusion limited myocardial injury. By analogy with the preconditioning phenomenon, this strategy applied following ischemia is known as ischemic postconditioning (Figure 1) and has been described in humans. Indeed, in patients with ongoing acute myocardial infarction, necrosis size has been significantly reduced by 36% using sequential re-inflation of the intracoronary balloon upstream of the stenting zone (13). Moreover, postconditioning by angioplasty has shown better long-term benefits in patients with acute myocardial infarction, including a reduction in infarct size at 6 months and an improvement in myocardial contractile function at 1 year (14).

**Figure 2:**
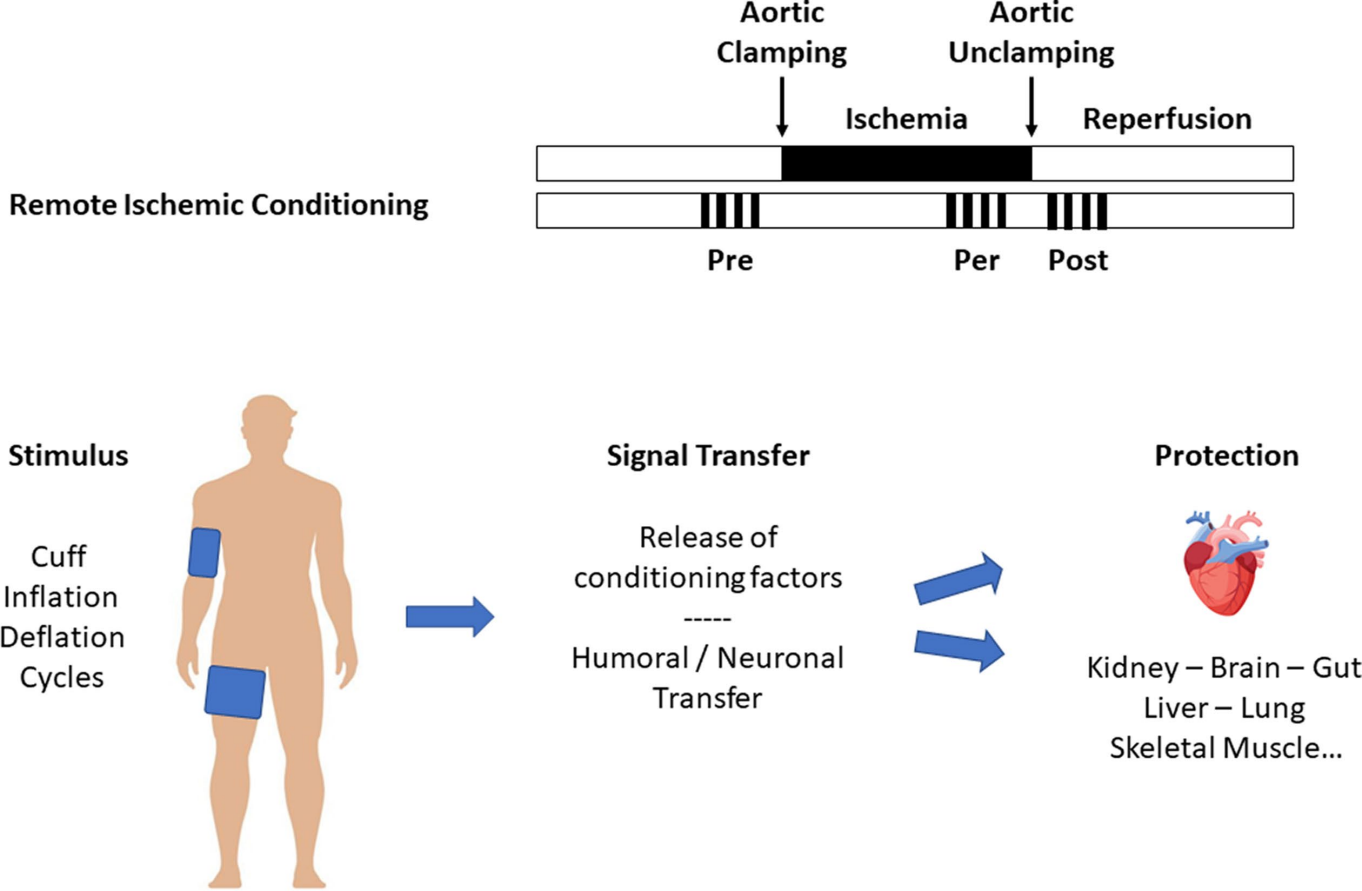
Remote ischemic conditioning.

**Figure 3:**
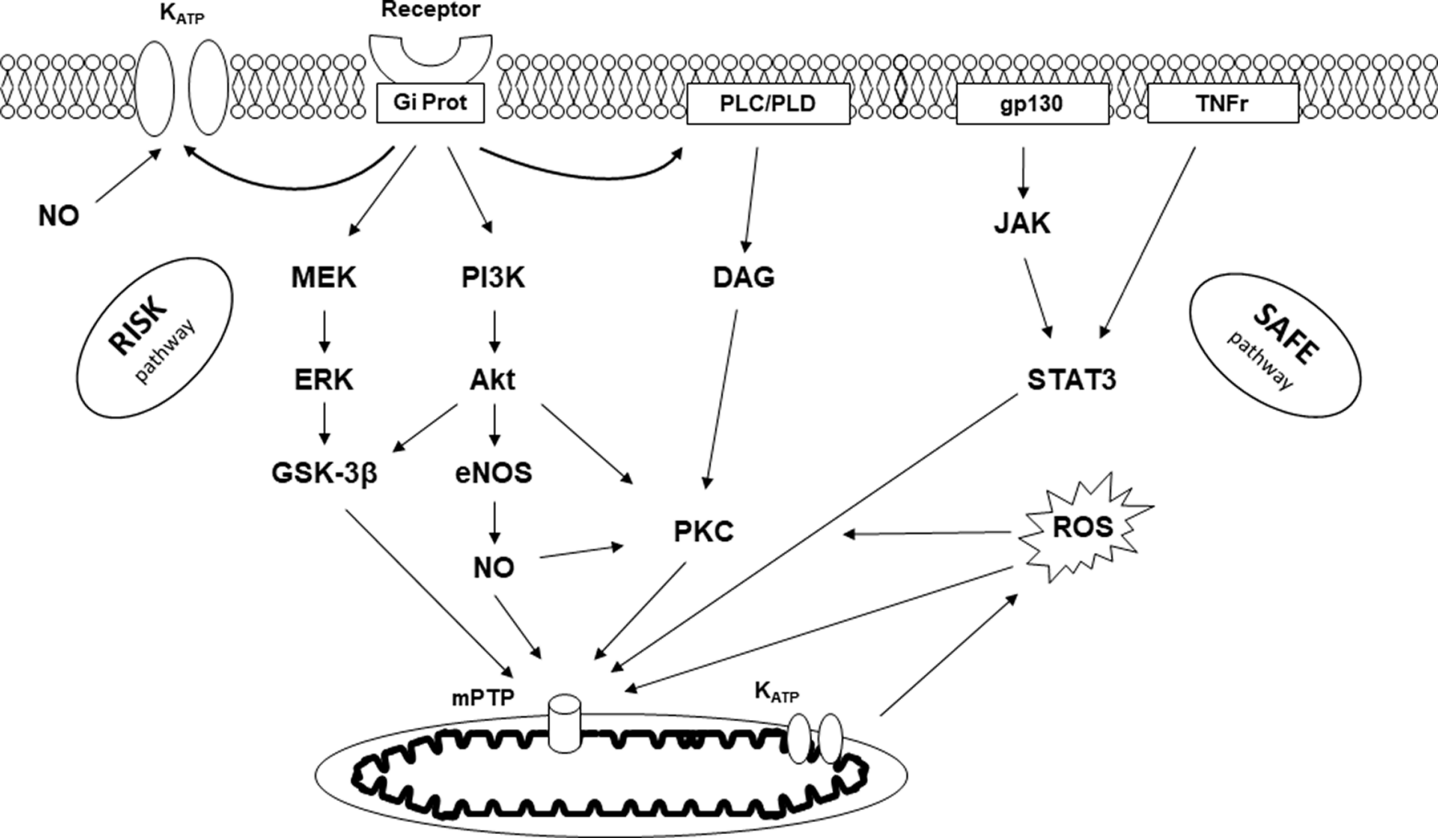
Signaling pathway of conditioning. K_ATP_, ATP-dependent potassium channel; Gi Prot, inhibitory G protein; PLC/PLD, phospholipase C/D; gp130, glycoprotein 130; TNFr, tumor necrosis factor receptor; RISK, reperfusion injury salvage kinase; SAFE, survivor activating factor enhancement; MEK, mitogen-activated protein kinase; ERK, extracellular signal-regulated kinase; GSK-3β, glycogen synthase kinase-3β; PI3K, phosphatidylinositol-3-kinase; Akt, protein kinase B; NO, nitric oxide; eNOS, endothelial NO synthase; DAG, diacylglycerol; PKC, protein kinase C; JAK, Janus kinase; STAT3, signal transducer and activator of transcription 3; ROS, radical oxygen species; mPTP, mitochondrial permeability transition pore.

Preconditioning and postconditioning are ubiquitous processes that protect organs against I/R injury. Many organs other than the myocardium (such as the brain, lung, kidney, digestive tract, and skeletal muscle) respond to conditioning. We also know that the various protective signals are transmitted remotely within the body via neuronal and humoral pathways, a process known as remote ischemic preconditioning (RIPC, Figure 2) (15). Finally, preconditioning can protect an organ for up to 24–72 h before an ischemic episode. This phenomenon, known as the late phase of preconditioning, follows the activation of protein neo-syntheses such as nitric oxide (NO) synthases and cyclooxygenase-2 (COX-2) in response to the administration of a signal-inducing protection (16). A vast body of literature has been devoted to deciphering the cellular mechanisms of conditioning (17, 18). Experimental studies carried out on both *in vivo* and *in vitro* models (isolated perfused heart, cell culture preparation, etc.) clarified the more intimate mechanisms (Figure 3). In brief, the protective signal travels to the cellular level, involving various surface receptors (notably those coupled with inhibitory G proteins), leading to the production of diacylglycerol, the activation of different protein kinases (such as protein kinase C), and the opening of various channels, such as ATP-gated potassium channels (K_ATP_). This information will then trigger different protective cascades via the reperfusion injury salvage kinases (RISK) and survivor activating factor enhancement (SAFE) pathways to lead to intracellular mitochondrial effectors. In the event of I/R, those organelles are the site of membrane permeabilization, known as the mitochondrial permeability transition pore (mPTP) opening (19). Permeabilization induces mitochondrial swelling, which causes them to lose their capacity to produce ATP at the level of the respiratory chain, ultimately dislocating within the cytoplasm and releasing pro-apoptotic substances such as cytochrome C. Conditioning works through several mechanisms: (i) delaying the mPTP opening, in particular through its action on a matrix protein (cyclophilin D); (ii) modifying the ROS production at the level of the mitochondrial respiratory chain (inhibition of complex I); and (iii) acting at the mitochondria-associated membrane, junction zones between the mitochondria, and the sarcoplasmic reticulum (20–23).

## 3 Volatile halogenated agents-induced myocardial protection

### 3.1 Experimental approach to myocardial conditioning with volatile halogenated agents

Ischemic conditioning has little direct clinical application in cardiac surgery. Numerous experimental studies have therefore focused on identifying the mechanisms of conditioning to decrypt, mimic, or amplify them. Volatile halogenated agents (VHAs) are the typical pharmacological application of the concept of conditioning in the operating room (Figure 1). Following several studies published in the late 80s, three independent teams demonstrated in 1997 the preconditioning effect of VHA (24–26). Comparing total intravenous anesthesia to VHA on a rabbit model, they reported a significant cardioprotective effect of VHA depending on both adenosine receptors and protein kinase C (24) and found that temporary administration of isoflurane reduced infarct size in dogs (25). The sulfonylurea glyburide abolished VHA-induced cardioprotection, highlighting the role of K_ATP_ channels in this mechanism (25). Since then, numerous studies have shown that VHA, administered before ischemia, reduced the size of myocardial infarction (27). This protective effect persists for several hours, a phenomenon called VHA-induced late preconditioning or second window of preconditioning (16). Meanwhile, Tanaka et al. (28) showed that animals anesthetized for 120 min with isoflurane had more than a 40% reduction in myocardial infarct size. This process results from the neosynthesis of proteins such as COX-2 (28) and endothelial NO synthases (eNOS) in response to VHA administration (29). In the late 1990s, several studies demonstrating the postconditioning effect of VHA were published (30–32). On isolated perfused hearts or *in vivo* models, halothane, enflurane, isoflurane, sevoflurane, and desflurane reduced myocardial injury when applied only during reperfusion. Sevoflurane-induced protection was observed at one minimal alveolar concentration (MAC), with no additional benefit at a higher dose (32). Even after cardioplegia, sevoflurane and desflurane provided additional protection when administered at the early phase of reperfusion (33). Intriguingly, 2 min of administration during reperfusion provided maximum protection, whereas less protective effects were observed with longer administration (34). The administration of one MAC isoflurane started 3 min before coronary reperfusion and maintained for only the first 2 min of reperfusion reduced myocardial infarct size by 50%, a phenomenon mediated by the activation of phosphatidylinositol-3-kinase (PI3K) signal transduction (35). VHA administration at this point could have a preferential impact at the mitochondrial level, resulting in a temporary inhibition of respiration, depolarization, and mitochondrial pH, subsequently slowing the opening of mPTP during the first time of reperfusion (36). Thus, VHAs are able to induce both valuable preconditioning and postconditioning (27, 37). Importantly, any postconditioning treatment must be applied at the initial phase of reperfusion before irreversible damage occurs. If the intervention is delayed for even 10 min, the protection is lost (38). In other words, VHA administration must be started a few minutes before reperfusion (before aortic unclamping) and continued immediately afterward in order to have a full protective effect.

### 3.2 Clinical studies of volatile halogenated agents in cardiac surgery

As early as 1999, Belhomme et al. (39) demonstrated that the administration of 5 min of isoflurane before aortic clamping during CABG surgery induced the activation of protein kinase C (a decisive step in the protective signaling pathway) as well as a decrease in postoperative troponin I. Subsequently, several studies by de Hert et al. (40–42) found marked cardioprotective effects of sevoflurane compared to propofol, responsible for the improvement in postoperative myocardial function, lower postoperative troponin I release, and shorter stay in the intensive care unit and hospital. Sevoflurane (43) and desflurane (44) significantly reduced postoperative troponin release in off-pump CABG surgery. However, those initial encouraging results have not been confirmed by other teams (45–48). More recently, the large MYRIAD trial (49), prospectively including over 5,000 patients in 36 centers, failed to demonstrate a benefit of VHA on 1-year mortality compared to intravenous anesthesia: 2.8% versus 3.0% (RR 0.94 [95% CI: 0.69–1.29], *p* = 0.71).

Several points need to be discussed. Anesthetic preconditioning seems to be more effective following repeated administration spaced by washout periods than continuous administration before ischemia. Two administrations of sevoflurane at one MAC spaced for at least 10 min significantly reduced serum troponin T release compared to a single continuous administration in patients undergoing CABG (50). Similarly, better cardioprotection following the administration of two sequences of one MAC sevoflurane for 5 min interspersed by a 5-min washout was found when compared with a single administration before CPB (51). The VHA administration protocol during surgery plays certainly a key role in the success of perioperative conditioning. Thus, the administration of VHA throughout surgery, including CPB, provided better protection than administration only before or after CPB (52). This specific efficiency of VHA, when administered during CPB, could be related to their immunomodulatory effects (53, 54). Finally, those cardioprotective effects of VHA must also be linked to their potential beneficial renal effects. It was reported in patients scheduled for CABG surgery that 10 min administration of two MAC sevoflurane during CPB and before aortic clamping significantly decreased postoperative release of brain natriuretic peptide and plasma cystatin C, biochemical markers of myocardial contractile, and renal dysfunction (55). The 1-year follow-up of this last cohort showed a reduced incidence of late cardiac events after surgery (56). Among the many meta-analyses published on the topic, Uhlig et al. (57) reviewed 45 cardiac surgery clinical studies involving 4,840 patients and found a reduction in overall mortality with VHA (OR 0.55; 95% CI: 0.35–0.85), as well as a reduction in perioperative complications. Similar results have been reported when VHAs were used throughout the surgical procedure (OR 0.66 [95% CI: 0.49–0.89]) (58). Those meta-analyses included small and sometimes single-center studies (59). International guidelines are in favor of the use of VHA during cardiac surgery (60, 61).

## 4 Remote ischemic preconditioning

The heart and other organs can be protected against I/R by applying brief periods of non-lethal I/R sequences to remote tissues, a phenomenon called RIPC (15). The simplest way to perform RIPC is to repeatedly inflate above systolic blood pressure, a blood pressure cuff placed at the root of the upper and/or lower limb (Figure 2). Numerous experimental studies demonstrated that the protective signal was transmitted to other organs, including the heart, via humoral and neuronal pathways. In a proof-to-concept study, Hausenloy et al. (62) demonstrated that a RIPC protocol consisting of three 5-min cycles of upper limb ischemia significantly reduced the postoperative troponin T release in patients undergoing CABG surgery. Other studies subsequently confirmed these initial encouraging results (63, 64). Meanwhile, Zarbock et al. (65) investigated the benefit of RIPC on renal protection during CPB and found that RIPC reduced acute kidney injury by 15% in high-risk patients. In a follow-up study of the same cohort, they showed persistent renal protection at 90 days with an 18% absolute risk reduction of acute kidney injury (66). However, those promising results have not been confirmed by others. A cycle of 3 × 5-min cuff inflations to 200 mmHg separated by 5-min periods of cuff deflation did not reduce troponin release or other organ protection during cardiac surgery (67). More recently, two multicenter, prospective, randomized trials involving a large number of patients were unable to demonstrate any benefit of RIPC. The RIPHeart study (68) found that a RIPC protocol did not modify the rate of a composite endpoint (postoperative myocardial infarction, stroke, renal failure, and death within 90 days) in 1,385 patients undergoing cardiac surgery. Similarly, the ERICCA trial (69) included 1,612 patients and, using a similar design and endpoint, found no benefit of RIPC. Moreover, a protocol combining RIPC and postconditioning (four cycles of 5-min ischemia/5-min reperfusion applied before and after CPB) did not improve the outcome in 1,280 patients scheduled for cardiac surgery (70). Finally, meta-analyses confirmed that RIPC could reduce postoperative troponin release without clinical benefit to overall outcomes (71, 72).

Several factors are expected to interfere with the clinical effectiveness of RIPC. The algorithm is probably of major importance in terms of the number of cycles, the duration of each ischemic sequence, and the RIPC application site (arm and/or thigh) (73, 74). In an *ex vivo* mice Langendorff model, Johnsen et al. (73) found that four, six, or eight cycles were effective, while two were not. Ischemic cycles lasting 2 min or 5 min reduced infarct size, but 10 min abolished cardioprotection. In clinical studies, the majority of medical staff use a 5-min ischemia protocol. There may be a dose dependence on the RIPC protocol, as previously suggested (74). Increasing the power of the stimulus could also be more effective, as demonstrated in patients undergoing cardiac surgery and having received a RIPC protocol by simultaneous inflation of a balloon on both the arm and the thigh (75). Drugs administered during cardiac surgery may also interfere with the effectiveness of a RIPC protocol. Thus, nitrates (despite their intrinsic cardioprotective properties) can inhibit the beneficial effect of RIPC (75, 76). Nitrates could inhibit RIPC-induced cardioprotection by NO inhibiting afferent nerve conduction in the limb. In the ERIC-GTN study (76), intravenous infusion of nitrates during surgery abrogated RIPC protection. Anesthetic agents could also affect the cardioprotective effectiveness of RIPC. Kottenberg et al. (77) reported that RIPC reduced the postoperative troponin release after CABG surgery in patients receiving isoflurane but not propofol. This latest study can be interpreted in two ways. Either the RIPC protocol was insufficiently powerful to protect the myocardium under clinical conditions and required synergistic protection by isoflurane or propofol by itself negated the protective effect of RIPC. Indeed, experimental studies have shown that propofol abolishes desflurane-induced preconditioning and RIPC (78, 79). In a recent meta-analysis focusing on the renal protective effects of RIPC, the authors emphasized that RIPC’s beneficial effects were mainly found during anesthesia with VHA (80).

## 5 Glycemic balance

**Figure 4:**
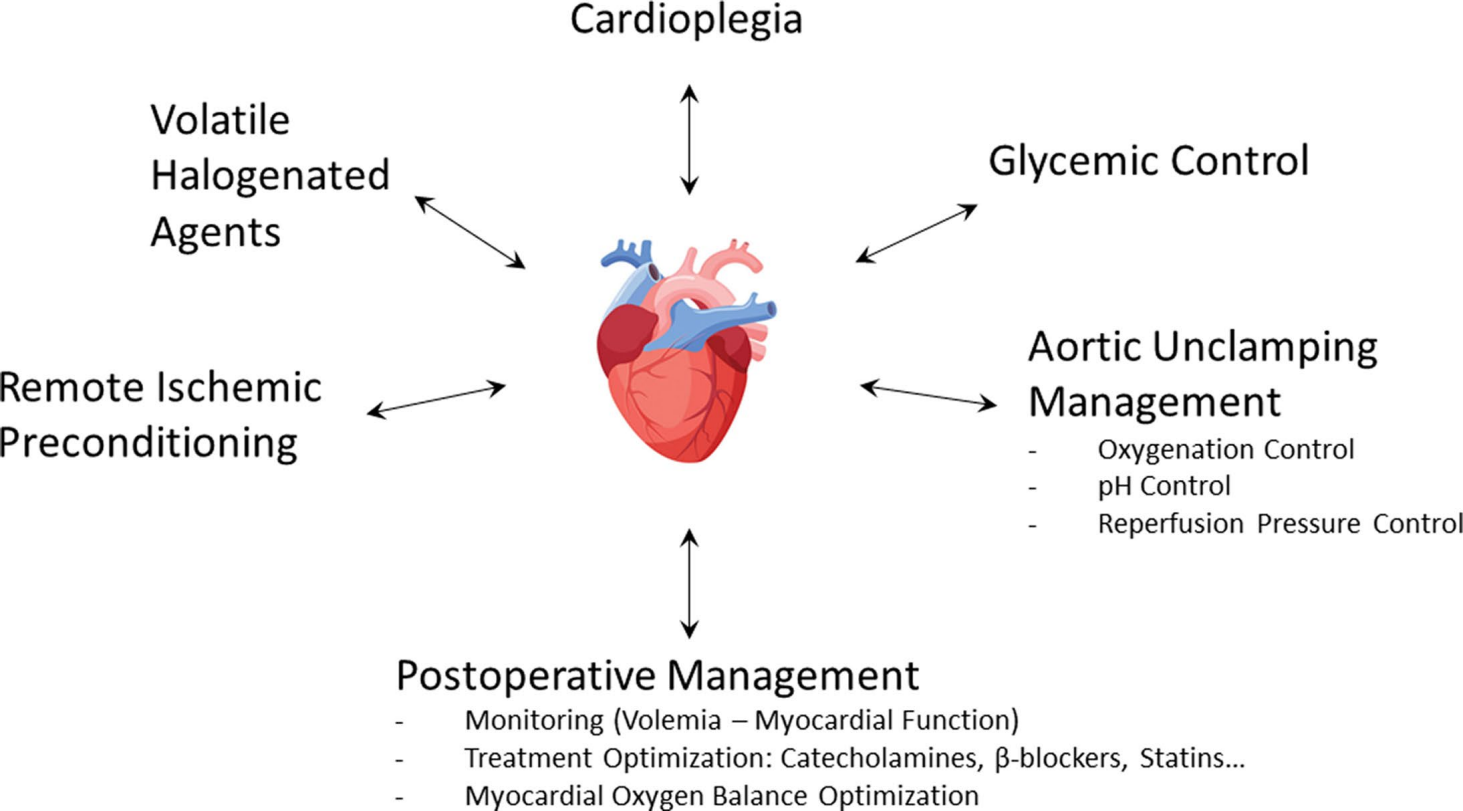
Bundle of care combining different cardioprotective interventions during cardiac surgery.

Hyperglycemia is both a common phenomenon observed during cardiac surgery and a well-known independent risk factor of mortality (81, 82). In a large cohort including more than 8,000 patients undergoing cardiac surgery, Ascione et al. (83) found that 15% of them had blood sugar levels above 200 mg/dL, more than half of perioperative hyperglycemia occurring in non-diabetic patients. Hyperglycemia was associated with postoperative myocardial infarction (OR: 2.73 [95% CI, 1.74–4.26]). Among various processes, acute hyperglycemia increases ROS production, leading to endothelial dysfunction and worsening of myocardial I/R injury (84–86). Experimentally, myocardial infarct size was linearly related to blood glucose concentration (87). Several studies have also demonstrated that cardioprotective strategies such as VHA-induced preconditioning and postconditioning were abolished in hyperglycemic conditions (88–90). In addition, oral antidiabetic drugs such as glyburide could also inhibit preconditioning (25). Insulin is therefore a first-line therapy during cardiac surgery. In addition to lowering blood glucose levels, insulin has cardioprotective properties by activating the RISK pathway, especially when administered during reperfusion (91–93). It is important to emphasize the benefit of continuous infusion of insulin rather than boluses (94, 95). Beyond hyperglycemia, the variability of blood glucose concentration is harmful (96). If hyperglycemia must be undoubtedly treated (97), the current objective is to determine the optimal blood glucose threshold to be reached during cardiac surgery. Several studies reported an increased incidence of stroke (98) and delirium (99) during intraoperative tight glucose control. In a retrospective study of 4,000 patients treated at the Cleveland Clinic, Duncan et al. (100) reported that maintaining intraoperative blood glucose levels below 140 mg/dL increased morbidity and mortality. However, the same team conducted a prospective study in which a treated group received a fixed high-dose insulin and concomitant variable glucose infusion during cardiac surgery (101). In more than 1,400 randomized patients, they observed a 38% reduction of 30-day morbimortality in the treated group, demonstrating once again the intraoperative protective effect of insulin. The current consensus is, therefore, to treat with continuous insulin infusion when intraoperative blood glucose values are 180 mg/dL (10 mM) or higher; the target range is between 140 and 180 mg/dL (7.7–10 mM) (102–106). Finally, glycemic management appears to be an integral part of the cardioprotection strategy during cardiac surgery (Figure 4).

## 6 The challenge of aortic unclamping

### 6.1 Oxygenation control

Uncoupling of the mitochondrial respiratory chain during ischemia induces ROS overproduction in the event of excessive oxygenation at reperfusion (107). *In vitro* and *in vivo* experiments demonstrated that hyperoxic reperfusion increased inflammatory response and apoptosis and altered hemodynamic performances (108). Additionally, hyperoxia causes a significant reduction in coronary blood flow, which can further exacerbate reperfusion injury (109). Several clinical studies emphasized the potentially harmful effects of high-concentration oxygen therapy for the treatment of heart disorders in the medical setting (110, 111). The AVOID trial (112) showed, in non-hypoxemic patients suffering from ST-segment elevation myocardial infarction, that high-flow O_2_ therapy at reperfusion increased the peak of creatine kinase and the rate of recurrent myocardial infarction. Similarly, a recent meta-analysis (113) did not find evidence to support the use of oxygen therapy in normoxemic patients with acute myocardial infarction. Although debated, those questions arise in the context of cardiac surgery with CPB (114–116). In pediatric heart surgery, controlled reoxygenation during CPB decreased the markers of inflammation and organ damage (117). In patients scheduled for CABG surgery, hyperoxia (PaO_2_ = 400 mmHg) compared to normoxia (PaO_2_ = 140 mmHg) during CPB increased oxidative myocardial damage (118). Inoue et al. (119) explored the effects of reducing hyperoxia (PaO_2_ = 450–550 mmHg versus 200–250 mmHg) during reperfusion in cardiac surgery. They found that lowering the reperfused PaO_2_ after aortic unclamping significantly reduced oxidative damage and myocardial enzyme release. Conversely, McGuinness et al. (120) failed to demonstrate any difference in myocardial ischemia damage when comparing modest hyperoxia (178 mmHg) with normoxia. Interestingly, there was significant pre- and post-CPB hyperoxia (approximately 200 mmHg) in both groups, precisely during high-risk periods of hyperoxia-induced cellular damage. It is therefore quite likely that hyperoxia at the time of aortic unclamping may worsen myocardial lesions. Moreover, intraoperative oxidative damage is associated with postoperative delirium and neuronal injury (121). To sum up, it seems reasonable to advise that excessive use of supplemental oxygen in normoxic patients, particularly at the time of aortic unclamping, could potentially lead to exacerbated myocardial injury (Figure 4).

### 6.2 Impact of acid–base balance

Using a pH electrode on a rabbit heart model, Cobbe et al. (122) demonstrated that tissue pH fell continuously during ischemia because of progressive H^+^ ion accumulation, with a rapid recovery of pH during the first minutes of reperfusion. However, it has been shown that part of reperfusion injury comes from the rapidity of the pH rise. In rat livers, a slow increase in pH over 15 min after reperfusion dramatically reduced LDH release, a phenomenon known as pH paradox (123). Some years later, this result was confirmed on a cardiomyocyte model (124). It is well established that sudden reperfusion generates ROS production and induces calcium overload (125). Rapid recovery of cellular acidosis during the first moment of reperfusion ultimately leads to hypercontraction of the myocardium. At this point, ROS production at the mitochondrial level induces mPTP opening and subsequent myocyte death, a phenomenon that can be prevented by initial acidotic reperfusion (126). Several techniques, such as hypercapnic acidosis and Na^+^/H^+^ exchange inhibition, used during reperfusion attenuate lethal damage, whereas an alkaline solution (pH = 7.6) aggravated it (127). How long this temporary acidosis should be maintained after reperfusion is probably a pivotal question. Using a Langendorff-isolated rat heart model, Ohashi et al. (128) found that reperfusion for less than 3 min with an acid solution provided better recovery. Experimentally, acid reperfusion for 3 min delays the normalization of myocardial tissue pH and enhances myocardial salvage (129). Several studies suggest that the protective effect of postconditioning could be mediated by prolonged transient acidosis (130–133). Experimentally, infusion of NaHCO3 during a postconditioning protocol abolished cardioprotection and blunted the activation of RISK pathways (130). It is postulated that postconditioning prevents mPTP opening by maintaining temporary acidosis during the first minutes of reperfusion (131). VHA could also induce postconditioning through inhibition of respiration, depolarization, and ultimately mitochondrial acidification upon reperfusion (36). A complementary approach to maintaining a temporary acidosis during reperfusion is to inhibit Na^+^/H^+^ exchange. Administration of cariporide reduced Na^+^ overload and contributed to H^+^ extrusion during reperfusion (134, 135). Interestingly, this process also leads to a drop in Ca^++^ level during reperfusion (136). This proof of concept was demonstrated in the prospective, multicenter EXPEDITION study (137), which included 5,761 patients undergoing high-risk CABG surgery. The administration of cariporide before, during, and after surgery significantly reduced the incidence of myocardial infarction from 18.9% in the placebo group to 14.4% in the treatment group. However, because of an increase in cerebrovascular events in the treated group, the clinical use of cariporide was halted. Surprisingly, the EXPEDITION study design required cariporide administration to be continued for 48 h after surgery, i.e., a potentially too long way from the time of myocardial reperfusion. To summarize, the myocardial tissue is subject to significant pH variations at the time of aortic unclamping. At the very least, the clinician should avoid any alkalosis just before and just after myocardial reperfusion, which is very likely to be deleterious at this stage (Figure 4).

### 6.3 Controlling reperfusion pressure

Reperfusion injury may also be the result of excess pressure in the first moments following aortic unclamping. Okamoto et al. (138) demonstrated that early gentle reperfusion (50 mmHg versus 80 mmHg during the first 20 min of reperfusion) limited the post-ischemic damage in animals subjected to 4 h of ischemia. Similarly, a staged reperfusion protocol in which the coronary perfusion pressure was maintained at 40% of control for 0–3 min after the onset of reperfusion, 60% of control for 4–6 min, and 80% of control for 7–10 min has been suggested (139). This graduated reperfusion could mitigate myocardial stunning via transient acidosis during early reperfusion. Controlled reperfusion decreases calcium deposition and increases both mitochondrial oxidative phosphorylation rate and myocardial ATP content (140). Furthermore, low-pressure reperfusion limited myocardial necrosis by inhibiting mPTP opening on an isolated Langendorff heart model (141). Low-pressure reperfusion appears to offer similar protection to that provided by postconditioning, both techniques involving the activation of the PI3K-mPTP pathway (142). Testing this concept of gradual reperfusion on a population of patients undergoing CABG surgery, other authors (143) found a significant regression of interstitial edema at 60 min reperfusion. Although it is well established that excessive pressure worsens myocardial damage during reperfusion, there is currently no precise scheme for gentle reperfusion that can be clinically applied. Consequently, the practical implementation of this concept varies widely from one center to another (144).

## 7 The postoperative period

During the postoperative period, several factors will also influence myocardial tolerance to cardiac surgery with CPB. Maintaining the right balance between myocardial oxygen supply and demand remains a key issue. A transfusion threshold adapted to the needs of the myocardium and resumption of treatments such as beta-blockers at an early stage is traditionally part of good practices. In addition, two other specific points are worth mentioning here. During cardiac surgery, catecholamines are widely used to prevent or treat low-cardiac output syndrome, depending on preoperative patients’ status, the complexity of the surgical procedure, and, above all, the physician’s decision (145, 146). However, those drugs should be used only as needed to maintain adequate organ perfusion (147). Because of their positive inotropic and/or chronotropic effects, the overuse of catecholamines can lead to cardiac arrhythmias and myocyte death. Studying a large cohort of patients undergoing conventional cardiac surgery, we found (148) that perioperative use of dobutamine, simply based on the clinical judgment of the anesthesiologist, increased postoperative major cardiac morbidity. Exploring the data of a national cohort of 6,005 consecutive cardiac surgery patients, Nielsen et al. (149) demonstrated that inotropic therapy was independently associated with short- and long-term postoperative myocardial infarction and death. It seems essential to carefully monitor the patients’ macrohemodynamic parameters to correctly assess volemia and cardiovascular function so that catecholamines can be judiciously used and discontinued as soon as possible. In addition, the pleiotropic beneficial effects of statins have been widely described, hence their widespread use in cardiac surgery patients (150, 151). Although the administration of statins in the preoperative phase of cardiac surgery has recently been shown to be ineffective, physicians must be aware that these drugs can cause a rebound effect (152–154). Postoperative statin withdrawal was an independent predictor of postoperative myocardial infarction after major vascular surgery (155). Initiation of statin treatment results in endothelial eNOS upregulation due to the inactivation of a Rho protein, which usually inhibits eNOS. In the case of statin chronic therapy discontinuation, there is an overshoot translocation and activation of Rho, causing downregulation of eNOS production below baseline levels (156).

## 8 Modulating factors

During the intraoperative period, due to the multiplicity of patient-related factors (comorbidities and treatments) as well as operative techniques (anesthetic agents, CPB, and cardioplegia), the efficacy of cardioprotective techniques is questionable. First, the phenomenon of cardiac conditioning is influenced by age, with drop-in effectiveness in elderly patients (157). Senescent myocardium is particularly sensitive to ischemia, probably due to metabolic degradation and impaired mitochondrial function (158, 159). A progressive loss of response to preconditioning was demonstrated by comparing three cohorts of 3-, 12-, and 20-month-old rats subjected to 1 or 3 cycles of ischemic preconditioning (160). It has also been shown that the protective effect of sevoflurane gradually disappeared with age (161). Second, the cardioprotective effects of estrogens and their possible interference with preconditioning have been regularly reported. Estrogens are thought to be protective via several mechanisms: activation of K_ATP_ channels, reduced leukocyte adhesion, ROS, and NO production, and reduced calcium influx during ischemia (162–166). In an experimental study, the injection of 17b-estradiol was shown to induce cardioprotection mediated by mitochondrial K_ATP_ channels, identical to that of ischemic preconditioning (162). It was also found under similar conditions that female mice were already protected and that ischemic preconditioning did not provide any additional protection (167). Myocardial infarct size was significantly smaller in female rabbits compared to male rabbits, and isoflurane did not provide any additional benefit (166). Third, many pharmacological agents used during surgery could interfere with perioperative cardioprotection (Table 1). Several reviews have focused on the cardioprotective effects of opioids (168–170). These agents act via activation of κ-, δ-, and/or even μ-opioid receptors, leading to protein kinase C activation and potentiation of K_ATP_ channels opening (171, 172). Propofol is known to abolish myocardial conditioning, possibly due to its ROS scavenger effects (79, 173). Ketamine inhibits ischemic preconditioning through its action on K_ATP_ channels (174). This pharmacological effect appears to be linked to the stereoselectivity of ketamine since the S(+)-form is neutral on both early and late preconditioning (175, 176). Lidocaine could also interfere with either anti-apoptotic cardioprotective or antagonist effects (177–179). A large prospective, comparative, randomized, multicenter study demonstrated that xenon, known to induce preconditioning and postconditioning (180, 181), significantly reduced postoperative troponin release in patients undergoing CABG surgery when compared to total intravenous anesthesia (182). Cyclosporine, in addition to its immunosuppressive properties, is a potent inhibitor of mPTP opening by preventing the calcium-induced interaction of cyclophilin D with a pore component (183). A single intravenous bolus of cyclosporine administered before CPB—as preconditioning (184)—or 10 min before aortic cross-unclamping—as postconditioning (185)—reduced the extent of myocardial injury in patients undergoing CABG or aortic valve surgery. However, clinical trials about the use of cyclosporine in cardiac surgery were interrupted following the publication of the large-scale multicenter CIRCUS study (186), which failed to demonstrate any improvement in patients with ST-segment elevation myocardial infarction.

**Table 1:** Positive and negative clinical modulators of myocardial protection by conditioning.

Factors inducing cardioprotection	Factors inhibiting cardioprotection
Volatile halogenated agents	Hyperglycemia
Insulin	Diabetes
Estrogens	Aging
Opioids	Sulfonylureas
Xenon	Hyperoxia
Cyclosporin A	Alkalosis
Nitric oxide	Nitrates
Statins	
Lidocaine	

## 9 Synergistic approaches

Experimental studies have suggested that a bundle of care could reinforce myocardial conditioning (187, 188). The interaction between several interventions to mitigate cardiac reperfusion injury has been shown between VHA and a postconditioning intervention (35). These results further suggest that the administration of 0.5 MAC isoflurane at reperfusion, a concentration that does not provide cardioprotection alone, reduces the threshold of ischemic postconditioning. Huhn et al. (89) showed that hyperglycemia abolished sevoflurane-induced postconditioning and that cyclosporine A reversed this loss of protection. The combination of RIPC with local ischemic postconditioning has previously been tested in ST-elevation myocardial infarction (189, 190). However, the efficacy of those multimodal interventions on surrogate markers of reperfusion injury, such as serum creatine kinase-MB isoenzyme for the RIPOST-MI study (189) or the salvage index by cardiac magnetic resonance imaging for the LIPSIA CONDITIONING study (190), is controversial. The ProCCard study (191) evaluated the relevance of a cardioprotective bundle of care during cardiac surgery with CPB. A total of 210 patients were randomized into a standard-of-care group and a treatment group simultaneously combining five modes of cardioprotection: sevoflurane administration, RIPC, tight intraoperative blood glucose control, induction of a moderate respiratory acidosis to prevent the pH paradox phenomenon, and gentle reperfusion to limit myocardial reperfusion injury (Figure 4). Unfortunately, the primary endpoint (the postoperative 72-h area under the curve of high-sensitivity cardiac troponin I) was not significantly modified: the mean ratio between control and treatment groups was 0.92; 95%CI: 0.71–1.21; *p* = 0.55. However, VHA administration modalities during CPB could be a crucial point in that study (192). These various factors indicate that the notion of synergy in cardioprotection remains a point that needs to be further explored.

## 10 Conclusion

After many years of translational research in the field of perioperative cardioprotection with inconsistent results, many questions remain. The specificity of the pathophysiology of I/R during cardiac surgery with CPB makes the equation complex. Notably, the myocardium is subjected to global ischemia on a non-beating heart, to which are added the effects of cardioplegia as well as those of CPB. This review highlighted different perioperative strategies to limit perioperative myocardial injury in patients undergoing cardiac surgery with CPB. Myocardial preconditioning and postconditioning, despite their limited clinical applications, have highlighted the understanding of the underlying mechanisms of intraoperative I/R myocardial injury. It is now up to the healthcare providers to integrate these different elements and maintain a comprehensive approach pre-, intra-, and post-CPB to limit intraoperative myocardial injury as effectively as possible during cardiac surgery.

## Author contributions

PC: Conceptualization, Validation, Writing – original draft. J-LF: Conceptualization, Supervision, Validation, Visualization, Writing – review & editing.
